# Editing of the *OsACS* locus alters phosphate deficiency-induced adaptive responses in rice seedlings

**DOI:** 10.1093/jxb/erz074

**Published:** 2019-02-27

**Authors:** Han Yong Lee, Zhixiong Chen, Cankui Zhang, Gyeong Mee Yoon

**Affiliations:** 1 Department of Botany and Plant Pathology, Purdue University, West Lafayette, IN, USA; 2 Center for Plant Biology, Purdue University, West Lafayette, IN, USA; 3 State Key Laboratory for Conservation and Utilization of Subtropical Agro-bioresources, South China Agricultural University, Guangzhou, Guangdong, China; 4 Department of Agronomy, Purdue University, West Lafayette, IN, USA

**Keywords:** Adaptive response, CRISPR/Cas, ethylene biosynthesis, *OsACS*, phosphate deficiency, rice

## Abstract

Phosphate (Pi) deficiency severely influences the growth and reproduction of plants. To cope with Pi deficiency, plants initiate morphological and biochemical adaptive responses upon sensing low Pi in the soil, and the plant hormone ethylene plays a crucial role during this process. However, how regulation of ethylene biosynthesis influences the Pi-induced adaptive responses remains unclear. Here, we determine the roles of rice 1-aminocyclopropane-1-carboxylic acid (ACC) synthase (ACS), the rate-limiting enzymes in ethylene biosynthesis, in response to Pi deficiency. Through analysis of tissue-specific expression of *OsACS* in response to Pi deficiency and *OsACS* mutants generated by CRISPR/Cas9 [clustered regularly interspaced short palindromic repeat (CRISPR)/CRISPR-associated protein 9] genome editing, we found that two members of the *OsACS* family, i.e. *OsACS1* and *OsACS2*, are involved but differed in their importance in controlling the remodeling of root system architecture, transcriptional regulation of Pi starvation-induced genes, and cellular phosphorus homeostasis. Interestingly, in contrast to the known inhibitory role of ethylene on root elongation, both *OsACS* mutants, especially *OsACS1*, almost fail to promote lateral root growth in response to Pi deficiency, demonstrating a stimulatory role for ethylene in lateral root development under Pi-deficient conditions. Together, this study provides new insights into the roles of ethylene in Pi deficiency response in rice seedlings and the isoform-specific function of *OsACS* genes in this process.

## Teaser

Of five functional ethylene biosynthesis genes in the rice genome, *OsACS1* plays a more important role in regulating Pi deficiency-responsive root architecture modulation, Pi uptake, and Pi starvation responses

## Introduction

Rice is an important staple crop that feeds more than half of the world’s population. However, the yield and sustainability of rice suffer significantly from low phosphorus stress due to a high phosphorus fixation rate and naturally low phosphorus content in its natural habitats (Raghothama, 1999; Gamuyao *et al.*, 2012). Phosphorus is an essential macro-element for a plant throughout the whole life cycle (Lopez-Arredondo *et al*., 2014). It serves as an important structural element of nucleic acids, energy currency ATP, and phospholipids (Raghothama, 1999; Lin *et al.*, 2009; Zhang *et al.*, 2014). Whereas a wide range of phosphorus forms exist in the soil, only inorganic phosphate anions (Pi) are exclusively assimilated by plants (Holford, 1997). However, the low mobility and solubility of Pi become a serious constraint, which affects the overall growth and reproduction of the plants. To cope with the low availability of Pi in the soil, higher plants, including rice, undergo significant morphological and biochemical changes through local and systemic low Pi-sensing mechanisms (Neumann, 2015). The most common morphological changes in response to Pi deficiency include the alteration of root system architecture, such as inhibited primary root growth, increase in lateral and secondary root growth (Williamson *et al.*, 2001; Perez-Torres *et al.*, 2008), and the enhancement of fine root structures (e.g. root hairs) through a local adaptive response (Forde and Lorenzo, 2001; Lynch and Brown, 2001; Williamson *et al.*, 2001; Linkohr *et al.*, 2002; Lopez-Bucio *et al*., 2002; Nacry *et al.*, 2005). Pi-deficient plants also induce the systemic transcriptional response of Pi starvation-induced genes that are involved in ethylene biosynthesis, Pi transportation, and Pi homeostasis in the shoots (Song and Liu, 2015). Accumulating studies indicate that plant hormones play a pivotal role in these adaptive responses by integrating low Pi signals into plants (Rouached *et al.*, 2010; Nagarajan and Smith, 2012; Kazan, 2013). Among plant hormones, the gaseous plant hormone ethylene has been considered as an important modulator for both local and systemic Pi starvation-induced responses (Neumann, 2015; Song and Liu, 2015), yet the underlying molecular mechanism remains unclear.

Ethylene is derived from the amino acid methionine via two intermediates, *S*-adenosyl-l-methionine (SAM) and 1-aminocyclopropane-1-carboxylic acid (ACC) (Yang and Hoffman, 1984). SAM synthetase converts methionine to SAM, which is subsequently converted to ACC by ACC synthase (ACS). ACC is then oxidized to ethylene by ACC oxidase (ACO). The conversion of SAM to ACC by ACS is generally considered as the rate-limiting step of the pathway. Numerous studies have demonstrated that ACS is the primary target for controlling ethylene production in response to developmental changes or various biotic and abiotic stresses (Argueso *et al.*, 2007). In higher plants, ACS is encoded by a multigene family, and Arabidopsis contains 12 *ACS* genes in the genome, only eight of which encode functional ACS proteins (Yamagami *et al.*, 2003). The eight functional ACS proteins are further classified into type-1, type-2, and type-3 ACS based on the phosphorylation sites in the C-terminal extension (Chae and Kieber, 2005). The type-1 ACS protein has a single calcium-dependent protein kinase (CDPK) phosphorylation site and three mitogen-activated protein kinase (MAPK) phosphorylation sites in the C-terminal region. The type-2 ACS protein has a single CDPK phosphorylation site and a unique regulatory motif called a Target of ETO1 (TOE) in the C-terminus. TOE is the binding site for ETHYLENE OVERPRODUCER1 (ETO1) and its two paralogs ETO1-Like1 (EOL1) and EOL2 E3 ligases (Wang *et al.*, 2004). ETO1/EOL1/EOL2 are BTB/TRP-containing E3 ligases that control the degradation of type-2 ACS proteins via the 26S proteasome (Wang *et al.*, 2004; Yoshida *et al.*, 2006). In contrast to both type-1 and type-2 ACS, type-3 ACS protein does not contain known regulatory sites or motifs, including phosphorylation sites (Chae and Kieber, 2005). The completion of the rice genome sequence has revealed that rice (*Oryza sativa* L.) also has a multigene family of *ACS* genes containing at least five putative members (*OsACS1*–*OsACS5*) (Zarembinski and Theologis, 1994). The sequence homology analysis with Arabidopsis ACS proteins showed that rice ACS proteins can also be classified into three types of ACS (OsACS2 for type-1; OsACS1 for type-2; and OsACS3 to 5 for type-3) in a similar manner to those in Arabidopsis (Yoshida *et al.*, 2006; Lee and Yoon, 2018).

A number of studies have demonstrated that Pi deficiency induces the modulation of ethylene biosynthesis or alters the sensitivity of plants to ethylene in many plant species, including rice (He *et al.*, 1992; Song *et al.*, 2016; Zhu *et al.*, 2016; Shukla *et al.*, 2017). Upon sensing low Pi in the soil, plant species undergo alterations for ethylene biosynthesis, though there is discrepancy as to whether the Pi deficiency stimulates or inhibits the biosynthesis of ethylene (Drew *et al.*, 1989; Borch *et al.*, 1999; Kim *et al.*, 2008; Roldan *et al.*, 2016). Nevertheless, the level of ethylene in plants is mostly regulated via altering the expression of *ACS* or *ACO* (Tsuchisaka and Theologis, 2004; Graham *et al.*, 2006; Hernandez *et al*., 2007; Thibaud *et al.*, 2010; Roldan *et al.*, 2013). Multiple studies have demonstrated that both local and systemic sensing of low Pi stress result in modulation of the transcript levels of *ACS* and *ACO*, thus affecting ethylene biosynthesis. For example, a transcript analysis of Pi-starved Arabidopsis roots has demonstrated that low Pi stress increases the transcripts of ethylene biosynthetic genes (Misson *et al.*, 2005; Thibaud *et al.*, 2010; Chacon-Lopez *et al*., 2011). Furthermore, a split-root transcriptome analysis of Arabidopsis under low Pi stress has shown that the expression of a subset of *ACO* genes increased via the local sensing of Pi stress (Thibaud *et al.*, 2010). Similarly, the systemic response to low Pi stress increases the abundance of *ACS* transcripts in Arabidopsis leaves, resulting in enhanced ethylene biosynthesis in this tissue (Misson *et al.*, 2005). A study has also shown that the increased transcript levels of *ACS* genes were readily reduced to the levels before Pi starvation upon provision of Pi (Morcuende *et al.*, 2007), suggesting the tight correlation between ethylene biosynthesis and Pi availability. While the Pi-induced transcriptional regulation of *ACS* genes is recognized, the role of different ACS isoforms in Pi deficiency remains to be determined.

In addition to the regulation of ethylene biosynthesis at the transcriptional level, the post-translational modification of ACS is an important alternative to modulate the levels of ethylene rapidly in response to various stresses (Chae and Kieber, 2005). A recent work from the analysis of the *hypersensitive to Pi starvation3* (*hsp3*) mutant (Wang *et al.*, 2012) has suggested that regulation of the protein stability of ACS plays a role in the adaptation of a plant in Pi stress. The molecular cloning of *hsp3* has revealed that the Pi-hypersensitive phenotype of *hsp3* results from a mutation in ETO1 E3 ligases. As ETO1 E3 ligase specifically targets type-2 ACS proteins for their degradation (Wang *et al.*, 2004), this study implies that the post-translational control of type-2 ACS plays a role in the Pi-mediated stress response. Studies from the various combination of Arabidopsis *acs* knockout mutants and the biochemical analysis of ACS proteins have shown that different ACS isoforms share an overlapping function, yet each ACS isoform possesses a unique role in a specific developmental or stress response (Yamagami *et al.*, 2003; Tsuchisaka *et al.*, 2009). For example, type-1 ACS isoforms have a role in responding to pathogen invasion through the stress-activated MAPK cascade (Liu and Zhang, 2004). Type-2 ACS has a primary role in controlling ethylene biosynthesis in the dark through the crosstalk with other plant hormones (Lee *et al.*, 2017). In contrast, type-3 ACS appears not to be involved in stress responses by itself, but rather it works co-operatively with other types of ACS through formation of a heterodimer (Lee *et al.*, 2017). Similar to Arabidopsis, rice possesses three different types of ACS isoforms (Yoshida *et al.*, 2006; Lee and Yoon, 2018). Interestingly, the transcript analysis of *OsACS* has demonstrated that only a subset of *OsACS* genes is differentially regulated under Pi-deficient conditions (Zhu *et al.*, 2016), indicating an isoform-specific role for *OsACS* genes in low Pi stress conditions. The existence of the ETO1-Like gene, *OsETOL1*, in the rice genomealso supports the possible type-specific role of *OsACS* in stress responses (Du *et al.*, 2014). Thus, it is of great interest to investigate the role of each isoform of *OsACS* in regulating Pi stress-induced adaptive responses.

In this study, we specifically investigate the role of *OsACS1* and *OsACS2* in Pi deficiency-induced adaptive responses in rice seedlings. Our results demonstrate that *OsACS1* and *OsACS2* are involved in several Pi deficiency-induced adaptive responses with a significant role in altering root system architecture. The results also show that *OsACS1* and *OsACS2* have an overlapping function in Pi starvation-induced responses, yet *OsACS1* plays a larger role in controlling Pi deficiency-induced adaptive responses, indicating the isoform-specific role of *OsACS* in this process.

## Materials and methods

### Plant materials and growth conditions

Rice (*Oryza sativa* L. cv. Nipponbare) seeds were dehusked and sterilized with 30% sodium hypochlorite for 10 min, followed by rinsing 5–6 times with deionized water. The sterilized seeds were subsequently placed onto solid Murashige and Skoog (MS) medium in a sterilized magenta box. The seedlings were grown in a growth chamber with a 16 h/8 h light/dark cycle at 28 °C. After 2 weeks, the seedlings were transferred to soil and grown in a greenhouse with a similar light/dark cycle and temperature conditions.

### Construction of recombinant DNA plasmid for clustered regularly interspaced short palindromic repeat (CRISPR)/CRISPR-associated protein 9 (Cas9)-mediated *OsACS* mutagenesis

A 20 nt guide RNA (gRNA) sequence for *OsACS1* and *OsACS2* was designed using CRISPR-PLANT (https://www.genome.arizona.edu/crispr/). Using an overlapping PCR, a fragment containing a rice U3 promoter and the gRNA for *OsACS1* or *OsACS2* was generated and subcloned into a plant binary vector, pARS3-MUb-Cas9, at the *Pme*I site using an infusion method. The final constructs were sequenced using the Sanger sequencing method.

### Rice transformation and selection

The rice transformation was performed by the *Agrobacterium tumefaciens*-mediated co-cultivation method, as described previously (Hiei *et al.*, 1994). T_0_ generation lines of Cr-*OsACS1* and Cr*-OsACS2* were selected on media with hygromycin, and the hygromycin-resistant T_0_ transgenic plants were analyzed to examine CRISPR/Cas9-induced mutations in the *OsACS* loci and the insertion of T-DNA containing the gRNA and the *Cas9* transgene using T7 endonuclease I (T7E1) analysis, PCR, and Sanger sequencing. The selected T_0_ lines with the *Cas9* transgene and mutations at the *OsACS* loci were self-pollinated to collect T_1_ seeds in a greenhouse with a 16 h/8 h light/dark cycle at 20 °C. The T_1_ plants were further analyzed to determine the zygosity of the mutation and the insertion of the *Cas9* transgene using the same method as described above.

### The identification of *OsACS* mutants by T7E1 and sequencing

T7E1 assay was conducted as previously described. PCR was performed by using gene-specific primers for each *OsACS* gene (Shan *et al.*, 2013; Wang *et al.*, 2014). To make heteroduplexes, the PCR product was reacted as follows: 95 °C for 10 min, 95–85 °C (ramp rate of –2 °C s^–1^), and 85–25 °C (ramp rate of –0.3 °C s^–1^). One unit of T7E1 was added to the PCR heteroduplexes and the reaction was incubated at 37 °C for 1 h. The T7 endonuclease-treated PCRs were separated by 2% agarose gel and visualized by ethidium bromide staining. Sequencing analyses were performed at the Purdue Genomics Core Facility by using ABI3730xl (Applied Biosystems™). Primers used are listed in Supplementary Table S1 at *JXB* online).

### Measurements of ethylene production

Ethylene measurements were performed as previously described (Hansen *et al.*, 2009) with a small modification. Surface-sterilized seeds were germinated in 22 ml GC vials containing 3 ml of MS with 1% sucrose and 1% bactoagar. After 2 d of dark treatment, the vials were capped and incubated at 28 °C for 3 d in the dark for ethylene measurement. For ethylene measurement of light-grown rice seedlings grown on either Pi-sufficient or -deficient media, capped vials were placed in a growth chamber with a 16 h/8 h light/dark cycle at 28 °C for 7 d. The accumulated ethylene was measured by GC using a Shimadzu GC2010 Plus capillary GC system with an HS-20 headspace autosampler. All treatments were measured from three vials each.

### RNA extraction and quantitative RT-PCR

Total RNA was extracted using an RNeasy Plus kit (Qiagen). cDNA was prepared from the total RNA using SuperScript II reverse transcriptase (Invitrogen) as described by the manufacturer. Quantitative real-time PCR (qRT-PCR) was performed using PowerUP™ SYBR^®^ green Master Mix (Applied Biosystems) and the CFX Connect™ Real-Time System (Bio-Rad). The relative expression of *OsACS* genes was normalized to the reference gene *OsActin1* and was calculated using the 2^ΔΔCT^ method. Primers used are listed in Supplementary Table S1.

### Hydroponic experiment

The modified hydroponic system was assembled as previously described (Negi *et al.*, 2016). Pi-sufficient (0.3 mM NaH_2_PO_4_) and Pi-deficient (0.015 mM NaH_2_PO_4_) media were prepared as previously described (Jain *et al.*, 2009) and buffered to pH 5.7 using 0.5 mM MES. Germinated seeds were placed onto sieves with Pi-sufficient or Pi-deficient media, and subsequently grown in a growth chamber with a 16 h/8 h light/dark cycle at 28 °C for 7 d.

### Soluble Pi content

Measurement of soluble Pi content was performed as previously described (Negi *et al.*, 2016). Rice plants were rinsed 5–6 times with deionized water and placed on 3MM paper for drying. Dried plants were ground in liquid nitrogen and further homogenized with 250 μl of 1% acetic acid, and vortexed for 1 min. After centrifugation at 10 000 rpm for 5 min, the supernatant was collected for assaying Pi content by using phosphomolybdate colorimetric assay as described (Ames, 1966).

## Results

### ACC promotes the alteration of rice root architecture in response to phosphorus

**Fig. 1.**
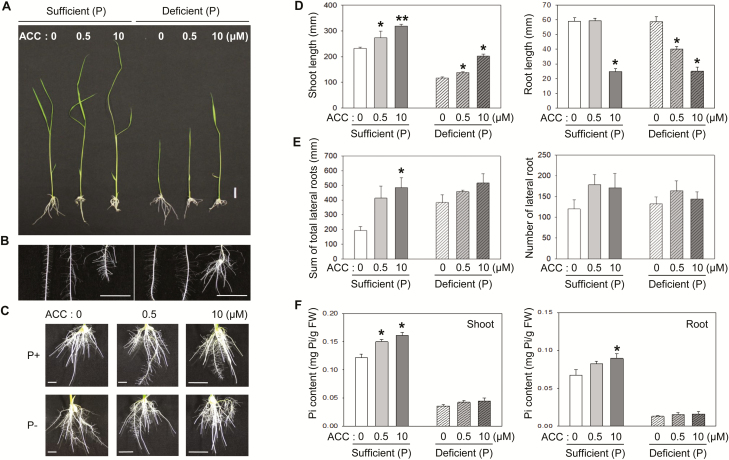
ACC accentuates the remodeling of root architecture in response to phosphorus. (A) Representative images of 7-day-old light-grown seedlings grown in a Pi-sufficient (0.3 mM) or -deficient (0.015 mM) condition in the presence of different concentrations of ACC. Scale bar=20 mm. (B, C) Representative images of the enlarged roots (B) or full root images (C) from seedlings in (A). Scale bars=10 mm. (D, E) Statistical analysis of the length of shoot and root (D) and total lateral root length and lateral root number (E). (F) Cellular Pi content of the shoot and root of rice seedlings grown in Pi+ or Pi– conditions with different ACC concentrations. Error bars indicate the SD; *n*=5, *P<0.05, **P<0.005, Student’s *t*-test.

Pi deficiency changes ethylene biosynthesis, resulting in the modulation of rice root architecture for better uptake of Pi in the soil (Peret *et al.*, 2014). To determine a dose effect of ethylene in relation to its function in controlling root architecture, wild-type (WT) rice seedlings were grown in hydroponic solutions supplemented with different concentrations of ACC, the direct precursor of ethylene (Adams and Yang, 1979), in either Pi-sufficient or -deficient conditions for 7 d. Longer lateral roots were observed in seedlings grown under Pi-deficient conditions than in those under Pi-sufficient conditions (Fig. 1B, C, E). Addition of ACC further accelerated the elongation of lateral roots regardless of the presence or absence of Pi, although the effect was dramatically enhanced in the Pi-sufficient plants (Fig. 1B, C); while the total lateral root lengths of both Pi-sufficient and -deficient seedlings were similar at the same concentration of ACC used in the growth medium, greater changes were found in the Pi-sufficient roots than in the Pi-deficient roots. Unlike the root length, ACC did not affect the total lateral root number from both conditions. Intriguingly, the primary root lengths of rice seedlings in either Pi-sufficient or -deficient conditions without ACC were comparable. However, addition of ACC led to an inhibited growth of primary root, and the primary root length was reduced in inverse relation to the increasing concentrations of ACC introduced in both the Pi-sufficient and -deficient media (Fig. 1A, C, D). Similar to the roots, we did not observe a prominent effect of ACC on the shoot growth of seedlings grown in Pi-deficient growth media compared with that of seedlings grown under Pi-sufficient conditions; Pi deficiency resulted in an inhibited shoot growth in seedlings grown in Pi-deficient media, yet seedlings in both growth conditions showed similar levels of enhancement in the shoot growth proportional to the increasing concentrations of ACC used in the growth media (Fig. 1A, D). Next, we examined the cellular Pi contents because ACC altered the root morphology of WT seedlings in both growth conditions (Fig. 1F). The Pi contents in both roots and shoots under the Pi-sufficient condition increased proportionally to the concentrations of ACC, indicating the positive role of ACC in Pi uptake. However, no obvious change in the Pi contents was found in the seedlings under Pi-deficient conditions. Taken together, these results indicate that ethylene plays a role in the alteration of shoot and root architecture, particularly lateral root length, and there is a positive correlation between the quantity of ethylene and the effect it exerts on the alteration of root morphology. The lack of response of the primary root to Pi deficiency may reflect the characteristic of a short-term induction period and/or the low ethylene content in the rice seedlings as the reduction in the primary root length of seedlings was correlated to the increasing dose of ACC used. A different response of primary root growth to Pi deficiency among different rice varieties could also be a reason for the lack of the primary root response in our study (Negi *et al.*, 2016).

### *OsACS1* and *OsACS2* are predominantly expressed in rice seedlings, and Pi deficiency leads to an increase in the transcript levels of both genes

**Fig. 2.**
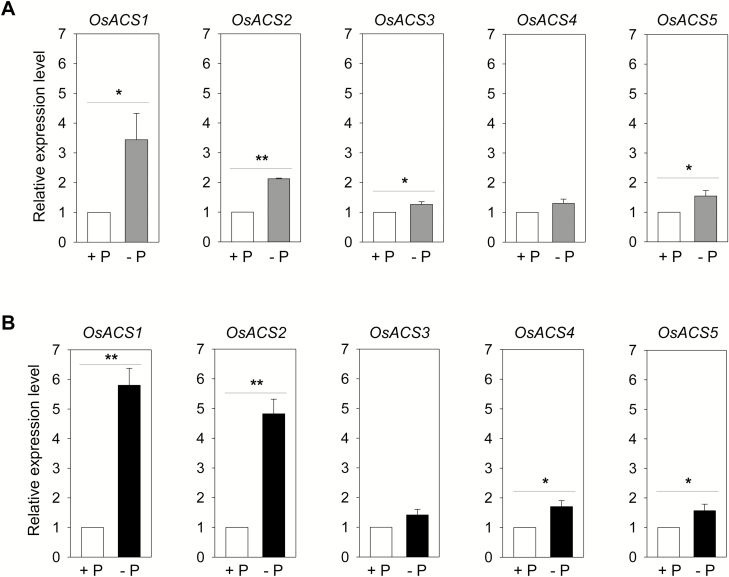
Transcript analysis of *OsACS* genes in light-grown rice seedlings in Pi-sufficient or -deficient conditions. (A, B) Relative gene expression of different *OsACS* genes in the shoots (A) or roots (B) in 7-day-old WT rice seedlings grown in the light. ‘+’ or ‘–’ indicate Pi-sufficient or Pi-deficient conditions, respectively. Error bars indicate the SD of three biological replicates. *P<0.05, **P<0.005, Student’s *t*-test.

The additive effect of ACC on the morphological changes in rice seedlings in response to phosphorus (Fig. 1) suggests that ACS is likely to play a role in this process because ACS controls the rate of ethylene biosynthesis through controlling the formation of ACC. The transcriptional regulation of ACS is the primary mechanism to control ethylene biosynthesis under many stress conditions in several plant species (Argueso *et al.*, 2007). To investigate whether Pi deficiency affects the expression of *OsACS* genes, the transcript levels of five *OsACS* genes from 7-day-old WT rice seedlings grown under Pi-sufficient or -deficient conditions were examined. The majority of *OsACS* genes, particularly *OsACS1* and *OsACS2*, were predominantly expressed in the shoots, except *OsACS3* under Pi-sufficient growth conditions (Supplementary Fig. S1). Similar transcript levels of *OsACS1*, *2*, *3*, and *4* were found in the root, but almost no expression of *OsACS5* was detected in this tissue (Supplementary Fig. S1). Pi deficiency significantly increased the transcript levels of *OsACS1* and *2*, but only led to a marginal or no increase in the transcript levels of the rest of the *OsACS* genes in both root and shoot tissues (Fig. 2). Moreover, the induction of the *OsACS1* and *2* transcripts in the root was ~1.7–2.4 times higher than that in the shoot under Pi deficiency. Taken together, these results suggest that the regulation of ethylene biosynthesis via *OsACS* genes, particularly *OsACS1* and *2* in the root, is probably one of the strategies that rice plants use to cope with Pi-deficient stress conditions.

### Generation of *OsACS1* and *OsACS2* mutants using CRISPR/Cas9-based genome editing

**Fig. 3.**
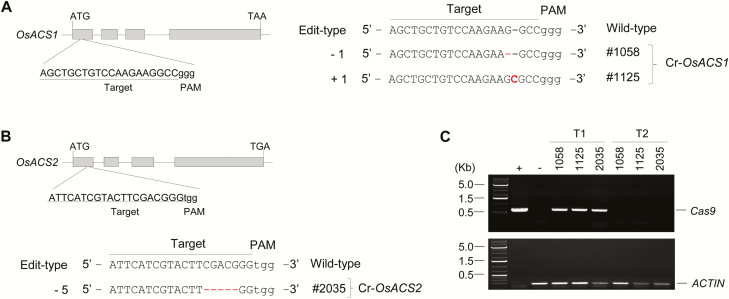
CRISPR/Cas9-based mutagenesis on *OsACS1* or *OsACS2*. (A, B) Schematic gene structure of *OsACS1* (A) or *OsACS2* (B) and detection of targeted mutation(s) (deletion or insertion) in the *OsACS1* (A) or *OsACS2* (B) locus. The target site nucleotides are shown in upper case letters and the protospacer adjacent motif (PAM) is indicated as lower case letters. ‘–’ and ‘+’ indicate either the deletion or insertion of nucleotide(s), respectively. (C) PCR-based identification of Cas-free *OsACS1* (Cr-*OsACS1*) and *OsACS2* (Cr-*OsACS2*) mutant plants. The gel image shows the presence or absence of *Cas* in the genomic DNA of T_1_ and T_2_ lines of Cr-*OsACS1* and Cr-*OsACS2*. ‘+’ indicates a positive plasmid control used in this study containing cloned *Cas9*. ‘–’ indicates a negative control genomic DNA isolated from WT rice.

To investigate further the role of ethylene in rice under Pi deficiency, we generated knockout lines of *OsACS1* or *OsACS2* using the CRISPR/Cas-based genome editing method (Supplementary Fig. S3). Two independent homozygous *Cas9* transgene-free Cr-*OsACS1* mutants (#1058 and #1125) and one homozygous Cr-*OsACS2* mutant (#2035) were selected for further studies (Fig. 3). The two selected independent Cr-*OsACS1* mutants have either a 1 bp deletion or a 1 bp insertion at 3 bp upstream of the protospacer adjacent motif (PAM) sequence, which results in a premature stop codon in the first exon of the *OsACS1* (Supplementary Fig. S4). The selected Cr-*OsACS2* has a 5 bp deletion at 2 bp upstream of the PAM sequence (Supplementary Fig. S4), resulting in a premature stop codon in the first exon of *OsACS2*. The off-target effects of the editing of *OsACS1* and *OsACS2* were examined by identifying potential off-target sequences using CRISPR-P v.20 (http://crispr.hzau.edu.cn/cgi-bin/CRISPR2/CRISPR). The top three highest potential off-target candidates of each *OsACS* gRNA were selected and examined using PCR amplification and Sanger sequencing. No mutations were found in the potential off-target loci in the Cr-*OsACS1* and Cr-*OsACS2* mutants (Supplementary Fig. S5).

### A mutation in *OsACS1* or *OsACS2* results in reduced ethylene biosynthesis and morphological changes in the etiolated rice seedlings

**Fig. 4.**
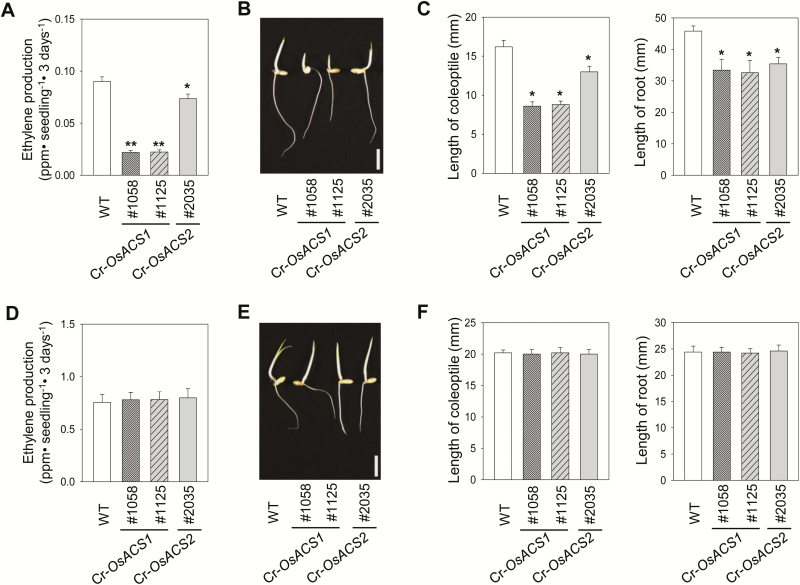
Morphological analysis of Cr-*OsACS* mutants and ethylene biosynthesis in the mutants. (A) Ethylene production of 3-day-old dark-grown WT and Cr-*OsACS* mutant seedlings. (B) A representative image of dark-grown Cr-*OsACS1* (#1058 and #1125) and Cr-*OsACS2* (#2035) mutant seedlings. Scale bar=10 mm. (C) Measurement of the coleoptiles and roots length of Cr-*OsACS1* and Cr-*OsACS2* mutant seedlings. Error bars indicate the SD; *n*=5; *P<0.05, Student’s *t*-test. (D) Ethylene production in dark-grown Cr-*OsACS* mutant seedlings grown in media supplemented with 10 µM ACC. (E) A representative image of dark-grown Cr-*OsACS1* and Cr-*OsACS2* mutant seedlings that were grown in a hydroponic solution containing 10 µM ACC. Scale bar indicates 10 mm. (F) Measurement of the coleoptiles and roots length of the mutant seedlings in the presence of 10 µM ACC. Error bars indicate the SD; *n*=5; *P<0.05, **P<0.005, Student’s *t*-test.

A successful disruption of ACS may cause a reduction of ethylene production in a plant as a result of a decrease in the concentration of ACC. We found that both Cr-*OsACS1* and Cr-*OsACS2* mutant lines produced less ethylene than WT seedlings in the dark (Fig. 4A). Interestingly, the levels of ethylene produced by two Cr-*OsACS1* mutants were significantly lower than the levels of ethylene produced in Cr-*OsACS2* (Fig. 4A), indicating a more important role for *OsACS1* in ethylene production than for *OsACS2* in the dark. The decreased ethylene biosynthesis in both Cr-*OsACS1* and Cr-*OsACS2* mutants was rescued by exogenously added ACC (Fig. 4D). This further confirms that the reduced levels of ethylene biosynthesis in both Cr-*OsACS* mutants were due to the inability of the mutants to produce a sufficient amount of ACC owing to the disruption of *OsACS1* or *OsACS2.*

The reduced ethylene biosynthesis in the Cr-*OsACS1* and Cr-*OsACS2* mutants appeared to affect the overall morphology of the mutant seedlings in the dark (Fig. 4B, C). Ethylene stimulates a coleoptile growth of rice seedlings in the dark. Therefore, examining the coleoptile growth is a good proxy for inferring defects associated with ethylene function in the mutants (Lee and Yoon, 2018). Both Cr-*OsACS1* and Cr-*OsACS2* mutants produced shorter coleoptiles than the WT in hydroponic solutions, although the phenotype is more rigorous in Cr*-OsACS1* plants. Additionally, Cr-*OsACS1* and Cr-*OsACS2* produced shorter roots compared with the WT, indicating the role of *OsACS1* and *2* in controlling root elongation. Similar to the ACC-mediated rescue of ethylene production in the Cr-*OsACS* mutants (Fig. 4D), ACC treatment rescued the growth inhibition on the coleoptiles and roots of the mutant seedlings (Fig. 4E, F). These phenotypes and the reduced ethylene production in the dark were successfully transmitted to the T_3_ generation in both Cr-*OsACS* mutants with the same mutation in the *OsACS* loci, corroborating the inheritability of the mutation by the next generation (Supplementary Figs S6, S7).

In addition to regulation of ethylene biosynthesis at the transcriptional level for *ACS* genes, our studies and those of others have demonstrated that controlling ACS protein stability via the interaction with other plant hormones is another strategy for regulating tissue ethylene content (Chae and Kieber, 2005; Argueso *et al.*, 2007; Yoon, 2015). For example, we have recently shown that cytokinin and brassinosteroid (BR) increase ethylene biosynthesis in etiolated rice seedlings without affecting transcript levels of all five *OsACS* genes (Lee and Yoon, 2018). As another proxy to confirm the disruption of *OsACS* function, ethylene biosynthesis in etiolated Cr*-OsACS* mutant seedlings in response to cytokinin and BR was measured (Supplementary Fig. S8). We found that unlike WT rice seedlings, Cr*-OsACS1* mutants, in which *OsACS2* is still active, almost completely failed to increase ethylene production in response to the treatment of cytokinin or BR. Different from the Cr*-OsACS1* mutant, cytokinin and BR slightly increased ethylene biosynthesis in the Cr-*OsACS2* mutant in which the *OsACS1* is still active, though the degree of increment was significantly smaller than that in WT seedlings. The lower reduction of ethylene biosynthesis in Cr*-OsACS2* in which OsACS1 protein (type-2 ACS) is presumably still functional aligns well with our previous studies demonstrating that type-2 ACS plays a predominant role in responding to cytokinin and BR for increasing ethylene biosynthesis (Lee *et al.*, 2017). These results associated with protein stability further support that CRISPR/Cas9-mediated mutagenesis successfully disrupted *OsACS1* and *OsACS2* genes, which inhibits cytokinin- or BR-induced ethylene biosynthesis due to the absence of functional OsACS1 or OsACS2 protein. We also confirmed that the T_3_ generation of both Cr-*OsACS* mutants showed reduced induction of ethylene biosynthesis in response to cytokinin (Supplementary Fig. S9). Together, these results demonstrate that the Cr-*OsACS* mutants generated by the CRISPR/Cas9-mediated targeted mutagenesis have a compromised *ACS* function and reduced ethylene content in rice, which led to morphological changes in the mutant seedlings.

### Lateral root elongation is decreased in Cr*-OsACS1* and Cr-*OsACS2* mutants under Pi-deficient conditions

**Fig. 5.**
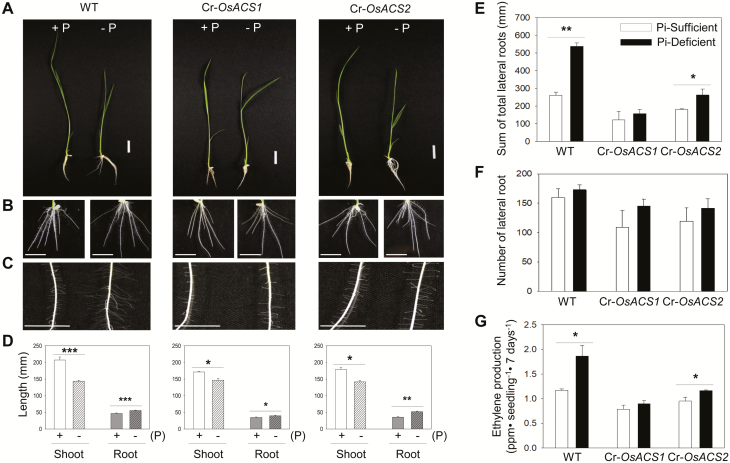
Altered morphologies and ethylene biosynthesis of light-grown Cr-*OsACS* mutants in Pi-sufficient or -deficient growth conditions. (A, B) Representative images of whole seedling of WT and Cr-*OsACS* mutants grown in either Pi-sufficient (+P) or -deficient (–P) conditions (A) and the enlarged root images of seedlings (B) in (A). Scale bars=20 mm. (C) Representative images of the enlarged lateral roots of WT and Cr-*OsACS* mutant seedlings in (A). Scale bars=10 mm. (D) The length of shoots and roots of WT and Cr-*OsACS* mutant seedlings grown in P-sufficient or -deficient conditions. (E, F) Measurement of the total lateral root length (E) and lateral root number (F) of WT and Cr-*OsACS* mutants grown in Pi-sufficient or -deficient conditions. (G) Pi deficiency-induced ethylene biosynthesis in the Cr-*OsACS* mutants. Seedlings were grown in either Pi-sufficient or -deficient media for 7 d and the accumulated ethylene was measured. Error bars indicate the SD; *n*=3, *P<0.05, **P<0.01, ***P<0.005, Student’s *t*-test.

Pi deficiency affects the overall architecture of a plant as part of the adaptive responses to cope with Pi limitation. Upon growth in Pi-deficient conditions, WT seedlings exhibited typical Pi deficiency-induced phenotypes which include inhibited shoot growth and an increase in secondary root and lateral root growth (Fig. 5A, E). To examine the roles of *OsACS1* and *OsACS2* in Pi deficiency-mediated morphological changes in rice, both Cr-*OsACS* mutant seedlings were grown under Pi-sufficient or -deficient conditions and their morphologies were examined. Since the two independent Cr-*OsACS1* mutant lines (#1058 and #1125) showed almost identical phenotypes and a similar reduction in ethylene production (Fig. 4), further studies were performed using only the #1058 mutant line for Cr-*OsACS1*. Under Pi-sufficient conditions, both Cr*-OsACS* mutants produced significantly shorter shoots (*P*<0.05) than WT plants (Fig. 5A, D; Supplementary Fig. S10). In addition, the growth of the primary root in both Cr-*OsACS* mutants was also inhibited (*P*<0.005) (Fig. 5A, D; Supplementary Fig. S10). Although both Cr-*OsACS* mutants displayed reduced shoot and root lengths compared with the WT in Pi-sufficient conditions, they still responded to Pi deficiency like the WT (Fig. 5D), resulting in shorter shoots and roots. It is worth noting that the degree of reduction in the shoot length was smaller in the Cr-*OSACS* mutants than in the WT. Under Pi-deficient conditions, both Cr-*OsACS* mutants displayed a significantly inhibited lateral root elongation in response to Pi deficiency (Fig. 5C, E). The Cr-*OsACS2* mutant showed a reduced sensitivity to Pi deficiency for lateral root elongation, but a slight increment of Pi-induced lateral root elongation was still observed (*P*<0.05) (Fig. 5E). Unlike the Cr-*OsACS2* mutant, the Cr-*OsACS1* mutant seedlings were almost unresponsive to Pi deficiency with regard to lateral root elongation (Fig. 5C, E). Although the induction of the lateral root elongation in Cr-*OsACS1* mutant seedlings was not statistically significant, the tendency for a slightly increased lateral root length in response to Pi deficiency indicated that *OsACS2* may also play a role in this process. In contrast to lateral root elongation, we did not observe differences in lateral root numbers between WT and both Cs-*OsACS* seedlings. These results suggest that the Pi deficiency-induced lateral root elongation is mainly a result of the overlapping function between *OsACS1* and *OsACS2*, although *OsACS1* plays a more significant role in this process.

### Light-grown Cr-*OsACS1* and Cr-*OsACS2* mutants have compromised ethylene biosynthesis in response to Pi deficiency

Pi deficiency results in an increase in ethylene production in several plant species, including rice, bean (*Phaseolus vulgaris*), white lupin (*Lupinus albus*), and *Medicago falcate* (Borch *et al.*, 1999; Gilbert *et al.*, 2000; Lin *et al.*, 2009). To examine the role of *OsACS1* and *OsACS2* in Pi deficiency-induced ethylene biosynthesis, the levels of ethylene in Cr-*OsACS1* and Cr-*OsACS2* mutants grown in Pi-sufficient or -deficient growth media with a 16 h/8 h light/dark cycle were measured. Consistent with previous studies, WT rice seedlings showed an enhanced ethylene biosynthesis under Pi-deficient conditions (*P*<0.05). However, the degrees of ethylene biosynthesis induced by Pi deficiency in both Cr-*OsACS1* and Cr-*OsACS2* mutants were considerably reduced, although the reduction was more dramatic in Cr*-OsACS1* (Fig. 5G). We found that the reduced ethylene biosynthesis in Cr-*OsACS* mutants is somewhat correlated to the reduced lateral root length in the mutant seedlings, indicating the positive relationship between lateral root elongation and ethylene biosynthesis in Pi deficiency. This result indicates that both *OsACS1* and *OsACS2* contribute to an enhanced ethylene production in rice seedlings in response to Pi deficiency.

### The expression of auxin-related genes is reduced in the roots of Cr-*OsACS* mutants

**Fig. 6.**
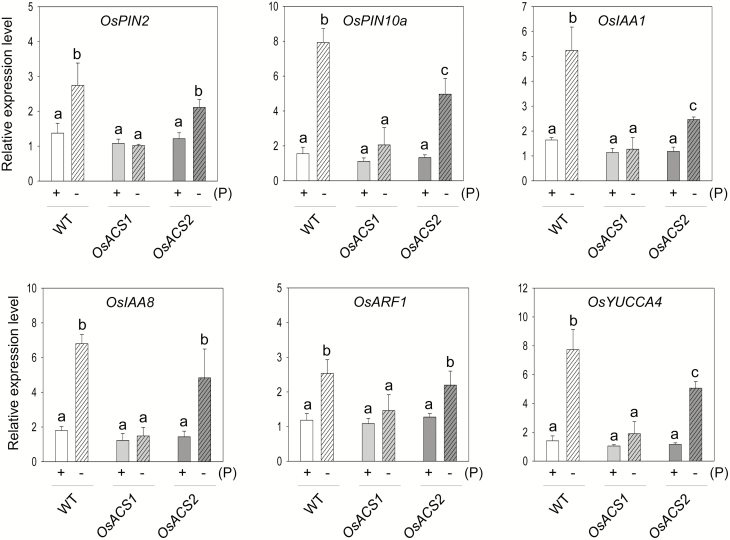
Pi deficiency-induced expression of auxin-related genes is altered in Cr-*OsACS* mutants. Seven-day-old light-grown seedlings were grown in either Pi-sufficient (+) or -deficient (–) conditions. *OsActin1* was used as an internal control. Error bars indicate the SD from three biological replicates. Different letters indicate a significant difference at *P*<0.05 (ANOVA, Tukey’s HSD post-hoc test).

The reduced total length of lateral roots in both Cr-*OsACS* mutants (Fig. 5) suggests that auxin might be involved in this process under Pi deficiency. To examine this, we determined the expression levels of a subset of auxin-related genes from auxin biosynthesis (*OsYUCCA4*), transport (*OsPIN2* and *OsPIN10a*), and signaling (*OsIAA1*, *OsIAA8*, and *OsARF1*), all of which have been shown to have increased expression in the roots of WT seedlings under Pi deficiency. As expected, Pi deficiency significantly induced the expression of all six genes in the WT roots (Fig. 6). The Cr-*OsACS2* mutant also showed increased expression of these genes to some extent, albeit at a minor level. In contrast, Cr-*OsACS1* almost failed to respond to low Pi stress to induce the expression of these genes. Taken together, these results indicate that auxin plays a role in lateral root elongation of rice seedlings via *OsACS1* and *2*.

### Pi homeostasis is perturbed in the Cr-*OsACS* mutants

**Fig. 7.**
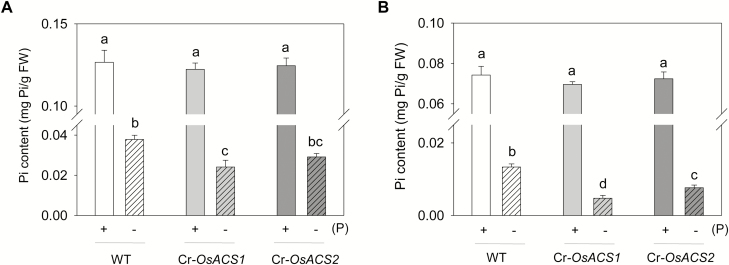
Cr-*OsACS* mutants have lower cellular Pi content than the wild type in Pi-deficient conditions. (A, B) Soluble cellular Pi contents in the shoots (A) or roots (B) of 7-day-old light-grown WT and Cr-*OsACS* mutants grown in Pi-sufficient (+P) or -deficient (–P) growth media. Error bars indicate the SD; *n*=3. Different letters indicate a significant difference at *P*<0.01 (ANOVA, Tukey’s HSD post-hoc test).

Under Pi-deficient conditions, rice alters root system architecture to ensure the efficient uptake of available soluble Pi from the soil. To examine the relationship between Pi deficiency-induced morphological changes and Pi uptake and distribution in the Cr-*OsACS* mutants, the soluble cellular Pi content in both Cr-*OsACS* mutants grown under Pi-sufficient or -deficient conditions was measured. Compared with the Pi-sufficient roots and shoots, the Pi content in the Pi-deficient shoots and roots of WT seedlings were significantly reduced (*P*<0.01) (Fig. 7). Under the Pi-sufficient conditions, the soluble Pi content in the shoots and roots of Cr-*OsACS* mutants was comparable with that in the WT. However, the shoot Pi content in the Cr-*OsACS1* mutant was significantly lower than that in WT seedlings after Pi deprivation for 7 d (Fig. 7A); a similar trend was observed in Cr*-OsACS2* although not statistically significant. Pi deprivation results in a significant reduction of Pi content in the roots of Cr-*OsACS1* and Cr*-OsACS2* mutants compared with that of WT seedlings, and the effect is more severe in Cr*-OsACS1*. Overall, these results demonstrate that *OsACS* genes, particularly *OsACS1*, are involved in regulating root architecture response and the associated Pi uptake and translocation.

### Expression of Pi deficiency-induced genes is down-regulated in the Cr-*OsACS1* and Cr-*OsACS2* mutants

**Fig. 8.**
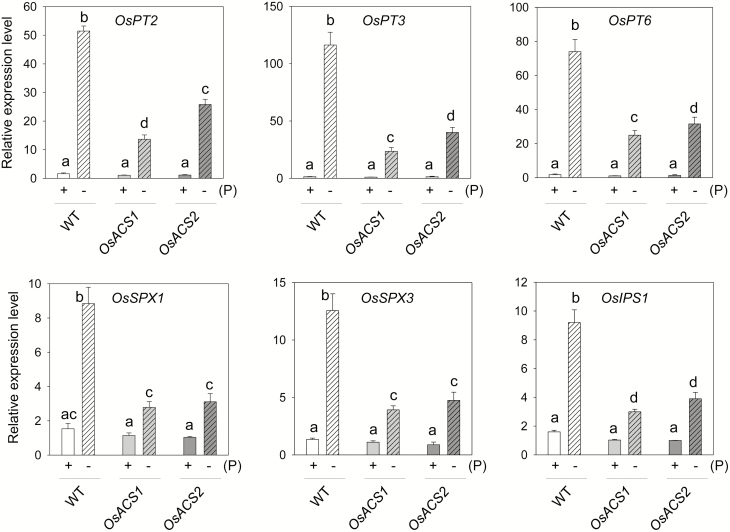
Cr-*OsACS* mutants exhibited a reduced expression of Pi deficiency-induced genes under low Pi stress. Seven-day-old light-grown seedlings were grown in either Pi-sufficient (+) or -deficient (–) media. *OsActin1* was used as an internal control. Error bars indicate the SD from three biological replicates. Different letters indicate a significant difference at *P*<0.05 (ANOVA, Tukey’s HSD post-hoc test).

The disturbed cellular Pi content in the Cr-*OsACS* mutants indicates that the transcriptional regulation of genes involved in the maintenance of Pi homeostasis may also change. To test this hypothesis, we examined the expression levels of a few representative Pi metabolism-related genes, namely those encoding three Pi transporter (*OsPT*), two SPX domain-containing protein (*OsSPX*), and one induced by phosphate starvation proteins (*OsIPS*). To this end, Cr-*OsACS* mutant seedlings were grown in Pi-sufficient or Pi-deficient conditions for 7 d. qRT-PCR results showed that both Cr-*OsACS1* and Cr-*OsACS2* expressed transcript levels of the six genes examined similar to those in WT seedlings under Pi-sufficient conditions. Under Pi-deficient conditions, the expression levels of the six genes in the WT seedlings substantially increased, which is consistent with previous studies (Zhou *et al.*, 2008; Ai *et al.*, 2009; Wang *et al.*, 2009; Wu *et al.*, 2013; Negi *et al.*, 2016) (Fig. 8). However, in contrast to the WT seedlings, both Cr-*OsACS* mutants, particularly Cr*-OsACS1*, showed a reduced sensitivity to Pi deficiency with respect to an increase in the transcript levels of most genes examined. For example, *OsPT2*, *OsPT3*, and *OsPT6* were induced by ~50- to 100-fold in the WT by Pi deficiency, but in Cr*-OsACS1* and Cr*-OsACS2*, the induction only ranged ~15- to 30-fold. Similar to the *OsPT* genes, Pi deficiency highly induced the expression of *OsSPX1*, *OsSPX3*, and *OsIPS1* genes in the WT, but not in CR-*OsACS1* and Cr-*OsACS2* mutants. Together, these results suggest that *OsACS1* and *2* are required for Pi acquisition to maintain Pi homeostasis via the induction of known Pi starvation-induced genes. Moreover, the greater decline in the Pi deficiency-induced expression of the *OsPT* genes in Cr-*OsACS1* suggests that *OsACS1* plays a more important role than *OsACS2* in the uptake and translocation of Pi under Pi-deficient conditions.

## Discussion

Lateral root elongation is one of the key changes that are induced upon the sensing of Pi deficiency (Sato and Miura, 2011). This branching of roots enables a plant to take up more Pi by increasing root surface areas and maximizing foraging of topsoil where Pi accumulates. The severe reduction on the lateral root elongation in both Cr-*OsACS* mutants in response to Pi deficiency indicates the positive effect of ethylene on root elongation. The stimulatory role of ethylene in lateral root elongation is intriguing as multiple studies have shown that ethylene generally inhibits root elongation during root development (Le *et al.*, 2001; De Cnodder *et al.*, 2005; Ruzicka *et al*., 2007). However, recent works have demonstrated a biphasic ethylene response in which ethylene has either a stimulatory or an inhibitory role in regulating root elongation depending on ethylene concentration and plant species (Pierik *et al.*, 2006; Pierik and Sasidharan, 2007). In sugar beet, a low concentration of ACC and the associated ethylene production promoted root elongation, while root growth was inhibited at high concentrations of ACC (Abts *et al.*, 2014). The biphasic mode of hormone response pertaining to plant growth regulation is not uncommon. BR or abscisic acid is known to either stimulate or inhibit root meristem size or root elongation, respectively, depending on its concentration (Prokic *et al.*, 2006; Gonzalez-Garcia *et al.*, 2011; Li *et al.*, 2017). Compared with the known inhibitory role of ethylene in lateral root initiation, the stimulatory role of ethylene in lateral root elongation has not been well described. One possibility for the stimulatory role of ethylene in lateral root elongation in response to Pi deficiency in rice seedlings is through a crosstalk with other plant hormones such as cytokinin. Cytokinin increases ethylene biosynthesis by stabilizing ACS proteins in Arabidopsis, and our recent studies showed that cytokinin probably plays a similar role in controlling ethylene levels in rice seedlings (Hansen *et al.*, 2009; Lee *et al.*, 2017; Lee and Yoon, 2018). A recent study has demonstrated that cytokinin stimulates lateral root elongation in rice (Rani Debi *et al.*, 2005). Together with the reduced auxin function in the mutants, this may explain the reduced lateral root elongation in both Cr-*OsACS* mutants in Pi-deficient growth condition as both mutants showed defects in enhancing ethylene levels in response to cytokinin treatment (Supplementary Figs S8, S9). Further studies on the crosstalk between ethylene and other phytohormones, including cytokinin, in the phosphate signaling pathway would shed light on Pi-induced lateral root development in rice.

The reduced expression levels of the selected *PSI* genes in Cr-*OsACS* mutants confirmed that *OsACS* is involved in the process. It also showed an *OsACS* isoform-specific role in the process because a greater reduction of *OsPT* genes was found in the Cr-*OsACS1* mutant. The reduction in the expression of *OsSPX* and *OsIPS* further suggests the potential role of ethylene in Pi sensing that is operated by a Pi concentration-dependent interaction between OsSPX1/2 and Phosphate Starvation Response Regulator 1 (OsPHR1) to control the expression of various *PSI* genes (Wang *et al.*, 2014). It would be of great interest to examine whether OsPHR1 is a direct target of the ethylene signaling pathway.

An intriguing observation from our studies is that *OsACS1* appears to play a larger role than *OsACS2*, while there are clear overlapping functions between two types of *OsACS* in controlling several Pi-induced adaptive responses. The disruption of *OsACS1* has more prominent effects on Pi deficiency-induced responses, such as inhibited lateral root elongation, reduced expression levels of Pi transport genes, and perturbed cellular Pi homeostasis. This might be related to the tissue- or cell type-specific gene expression characteristic of different isoforms of *OsACS* genes. We examined the gene expression of all five *OsACS* genes (*OsACS1*–*OsACS5*) in 7-day-old light-grown rice seedlings grown in either Pi-sufficient or -deficient growth conditions (Fig. 2; Supplementary Fig. S1). Among the *OsACS* genes examined, all the *OsACS* genes, except *OsACS3*, have higher expression in the shoot than in the root. A similar expression pattern was also found in 2-week-old rice seedlings, although higher expression of *OsACS* genes was generally found in the leaf blade (Supplemntary Fig. S2). There are no data available for *OsACS3* and *4* in RiceXPro, but the available microarray data for *OsACS1*, *2*, and *5* show that the expression of *OsACS1* and *2* is higher in roots than in shoots, while the expression of *OsACS5* remains high in the shoot. This suggests that the role of *OsACS1* and *2* is shifted from shoots to roots in mature rice (Sato and Miura, 2011). Despite the similar expression patterns among the *OsACS* genes in our study (Supplementary Figs S1, S2), only *OsACS1* and *2* were significantly induced under Pi deficiency and the changes in gene expression was more dramatic in the root than the shoot (Fig. 2). This is consistent with the general understanding that root architecture alteration in response to Pi deficiency is mainly regulated in roots rather than the systemic signaling from the shoot (Zhang *et al.*, 2014). The isoform-specific role of *OsACS* has been found in other stress conditions. For example, *OsACS1* and *OsACS5* are induced under submergence stress (Zarembinski and Theologis, 1997; Van Der Straeten *et al.*, 2001) and the transcript levels of *OsACS1* and *OsACS2* are up-regulated when rice plants are infected by *Magnaporthe oryzae* (Iwai *et al.*, 2006). The differential regulation of *OsACS1* and *OsACS2* in response to Pi deficiency can also be derived from regulation of different *ACS* transcription regulators. It has been shown that the disruption of MYB-type transcription factors PHR1 and its closest paralog PHL1, specifically down-regulated *ACS6* and *ACS7* in response to Pi deficiency (Bustos *et al.*, 2010).

Another possibility that could account for the larger role of *OsACS1* in Pi starvation-induced adaptive responses is the post-translational control of OsACS proteins such as protein stability. Previous studies and our recent studies on the protein stability regulation of Arabidopsis ACS proteins have demonstrated that input signals including plant hormones differentially regulate the stability of ACS in a type-specific manner (Lee *et al.*, 2017). For example, auxin rapidly enhances the protein stability of type-2 ACS, resulting in an increase in the steady-state levels of the protein by ~7-fold within 1 h of auxin treatment (Lee *et al.*, 2017). Auxin also extends the steady-state levels of type-1 ACS as well, yet it does not have any effect on type-3 ACS protein. The regulation of protein stability is one of the fastest ways for a plant to adapt to rapid developmental shifts or to respond to environmental stresses as it does not require gene expression. Pi deficiency is a severe environmental stress that requires the rapid adaptation of the plant to the surroundings, and thus it may require rapid mechanisms that initially enable the plant to sense the deficiency of Pi. A recent study on the Arabidopsis *hps3* mutant, which has a single amino acid substitution in ETO1 E3 ligase (Wang *et al.*, 2012), also supports the involvement of the post-translational control of ACS proteins in Pi deficiency-induced adaptive responses. This study underpinned the possible type-specific role of ACS proteins in Pi stress response because ETO1 E3 ligase specifically targets type-2 ACS proteins for degradation via the 26S proteasome. Further studies on the protein stability of OsACS proteins in relation to Pi deficiency will throw light on the roles of post-translational control of the OsACS proteins during Pi stress.

## Supplementary data

Supplementary data are available at *JXB* online.

Fig. S1. Transcript analysis of *OsACS* genes in light-grown seedlings under Pi-sufficient growth condition.

Fig. S2. Gene expression analysis of *OsACS* in 2-week-old rice seedlings.

Fig. S3. Schematic diagrams of the pARS-MUbCAS9-OsACS constructs used in this study.

Fig. S4. Sequencing results of the Cr-*OsACS* mutants.

Fig. S5. Sequencing results of the potential off-target sites.

Fig. S6. Morphological analysis of Cr-*OsACS* T3 mutants and ethylene biosynthesis in the mutants.

Fig. S7. Sequencing results of the T_3_ generation of Cr-*OsACS* mutants.

Fig. S8. Cr-*OsACS* mutants have impaired hormone-induced ethylene biosynthesis.

Fig. S9. T_3_ generation of Cr-*OsACS* mutants have impaired cytokinin-induced ethylene biosynthesis.

Fig. S10. Altered morphology of Cr-*OsACS* mutants in Pi-sufficient condition.

Table S1. List of the primers used in this study.

Supplementary Figures S1-S10

Supplementary Table S1
