# Supplementary material for: Editing of the OsACS locus alters phosphate deficiency-induced adaptive responses in rice seedlings
Source: J Exp Bot. 2019 Feb 27;70(6):1927–40. doi: 10.1093/jxb/erz074 (PMC6436150; doi:10.1093/jxb/erz074)
Supplement: Supplementary Figures S1-S10 [file erz074_suppl_supplementary_figures_s1-s10.pdf]

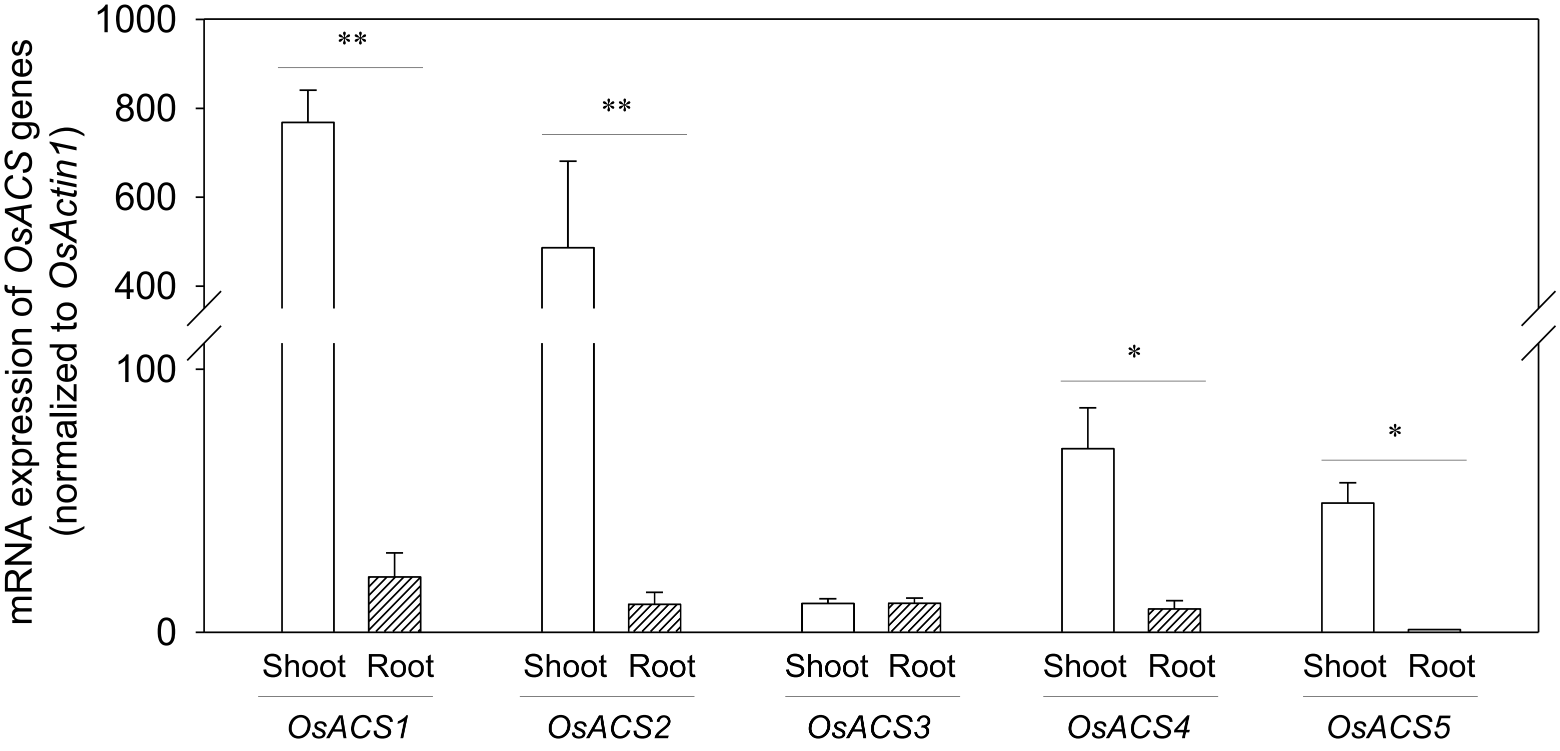

Fig. S1. Transcript analysis of *OsACS* genes in light-grown seedlings under Pi-sufficient growth condition. Wild-type rice seedlings were grown in Pi-sufficient solution for 7 days under the light and total RNA was separately extracted from shoots or roots from the seedlings. *OsActin1* was used as an internal control. Error bars indicate SD from three biological replicates. * $P < 0.05$, ** $P < 0.005$, Student's *t*-test.

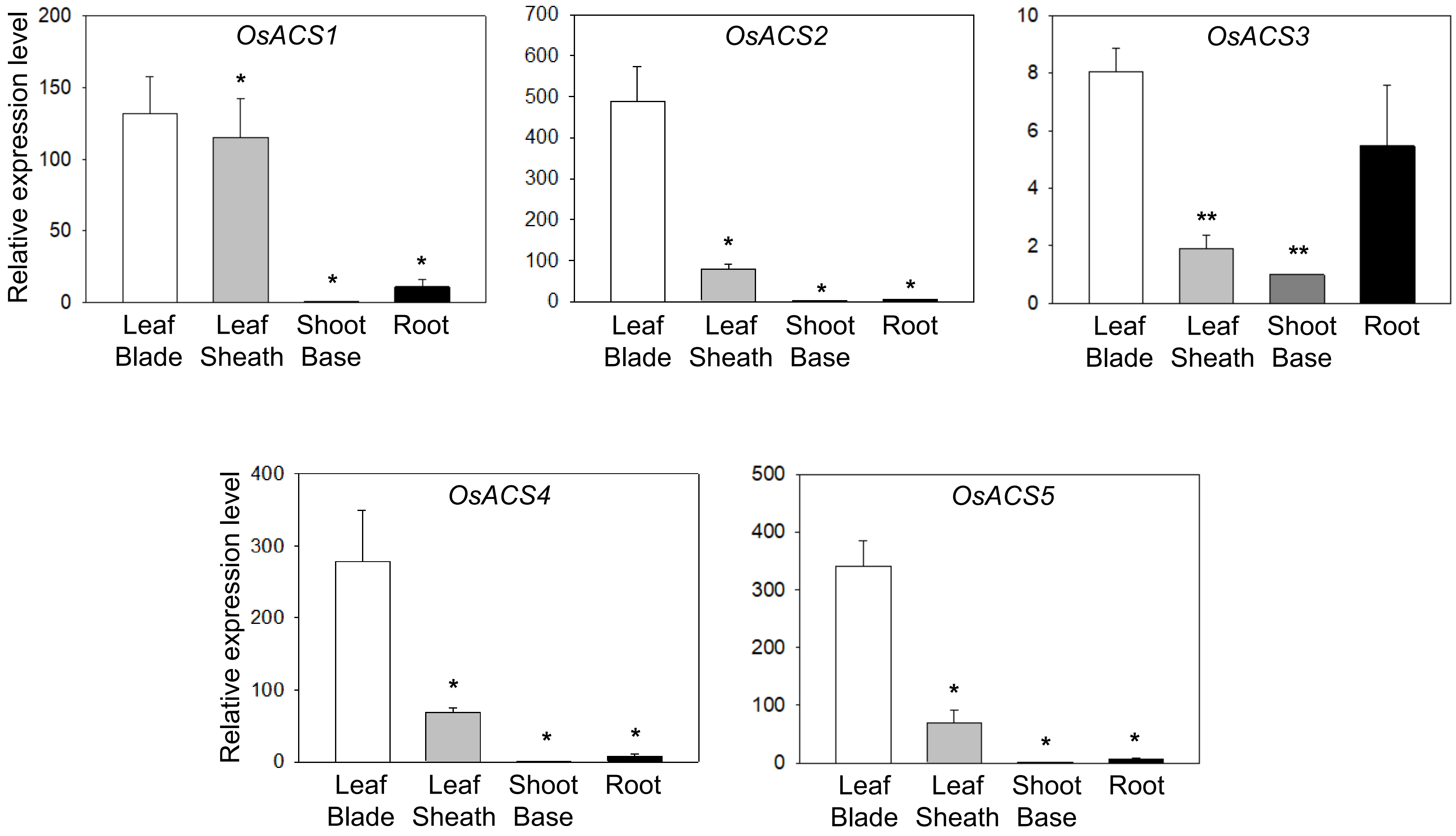

Fig. S2. Gene expression of *OsACS* in two-week-old rice seedlings. Two-week-old rice seedlings were grown hydroponically and total RNA were separately extracted from leaf blade, leaf sheath, shoot base and root. *OsActin1* was used as an internal control. Error bars indicate SD from three biological replicates. * $P < 0.05$, ** $P < 0.005$, Student's *t*-test.

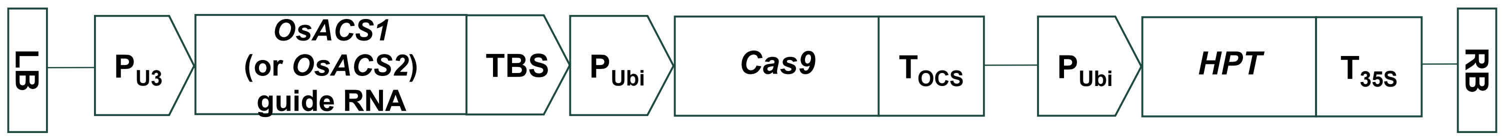

Fig. S3. Schematic diagrams of the pARS-MUbCAS9-OsACS constructs used in this study. The expression of the *OsACS1* or *OsACS2* guide RNA scaffold is driven by the rice U3 promoter (P_{U3}); The expression of Cas9 is driven by the maize ubiquitin promoter (P_{Ubi}); the expression of hygromycin (HPT) is driven by a maize ubiquitin promoter; TBS: transformation booster sequences; T: terminator; LB: left border; RB: right border.

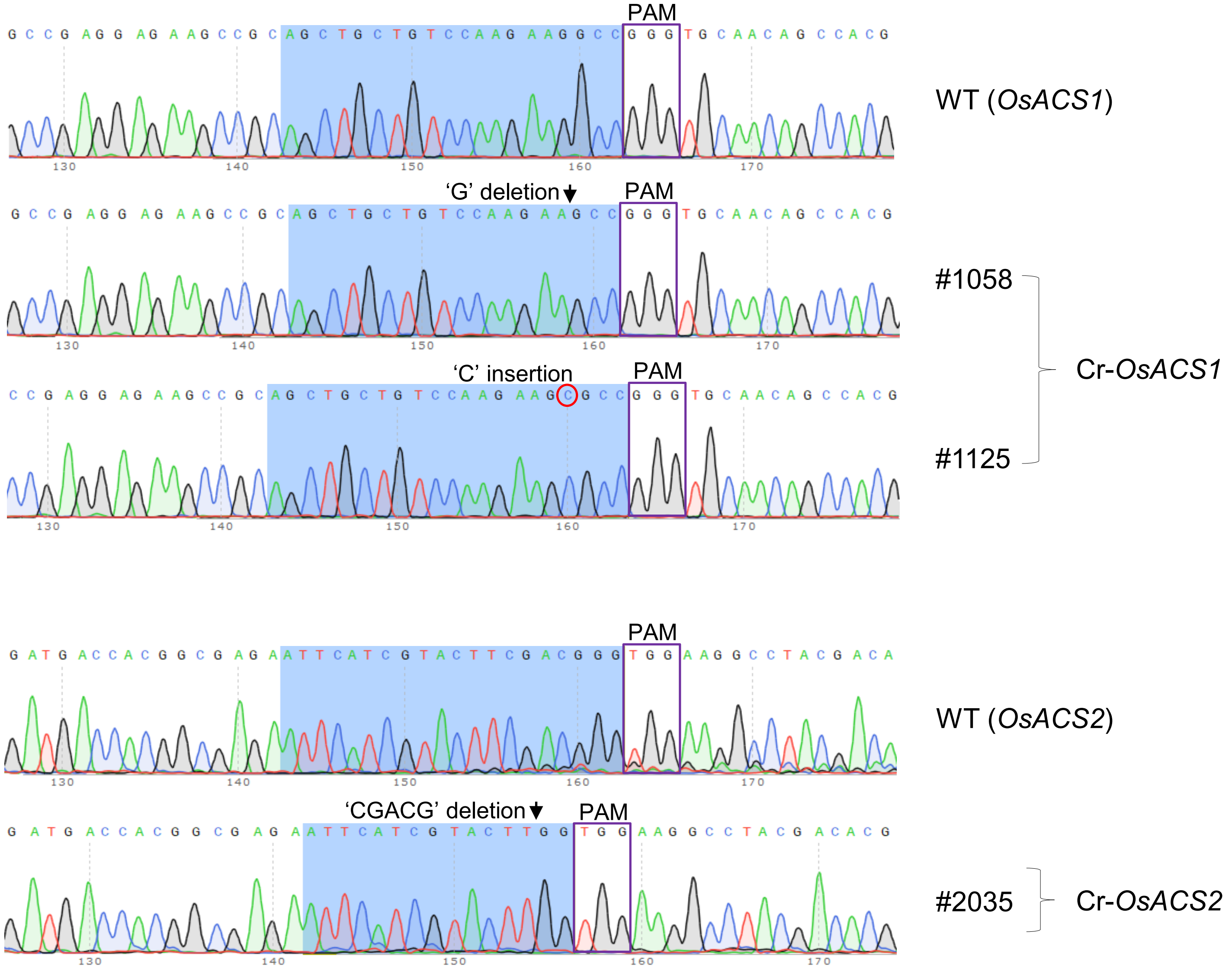

Fig. S4. Sequencing results of the Cr-*OsACS* mutants. The genomic DNA of wild-type and T2 Cr-*OsACS* mutants without *Cas* were used for Sanger-sequencing analysis. The regions spanning the target sites of *OsACS1* (#1058 and #1125) and *OsACS2* (#2035) were PCR amplified and sequenced. A 1-bp deletion or 1-bp insertion was detected in the Cr-*OsACS1* (#1058 and #1125, respectively). A 5-bp deletion was detected in the CR-*OsACS2* mutant (#2035). PAM sequences are squared with boxes.

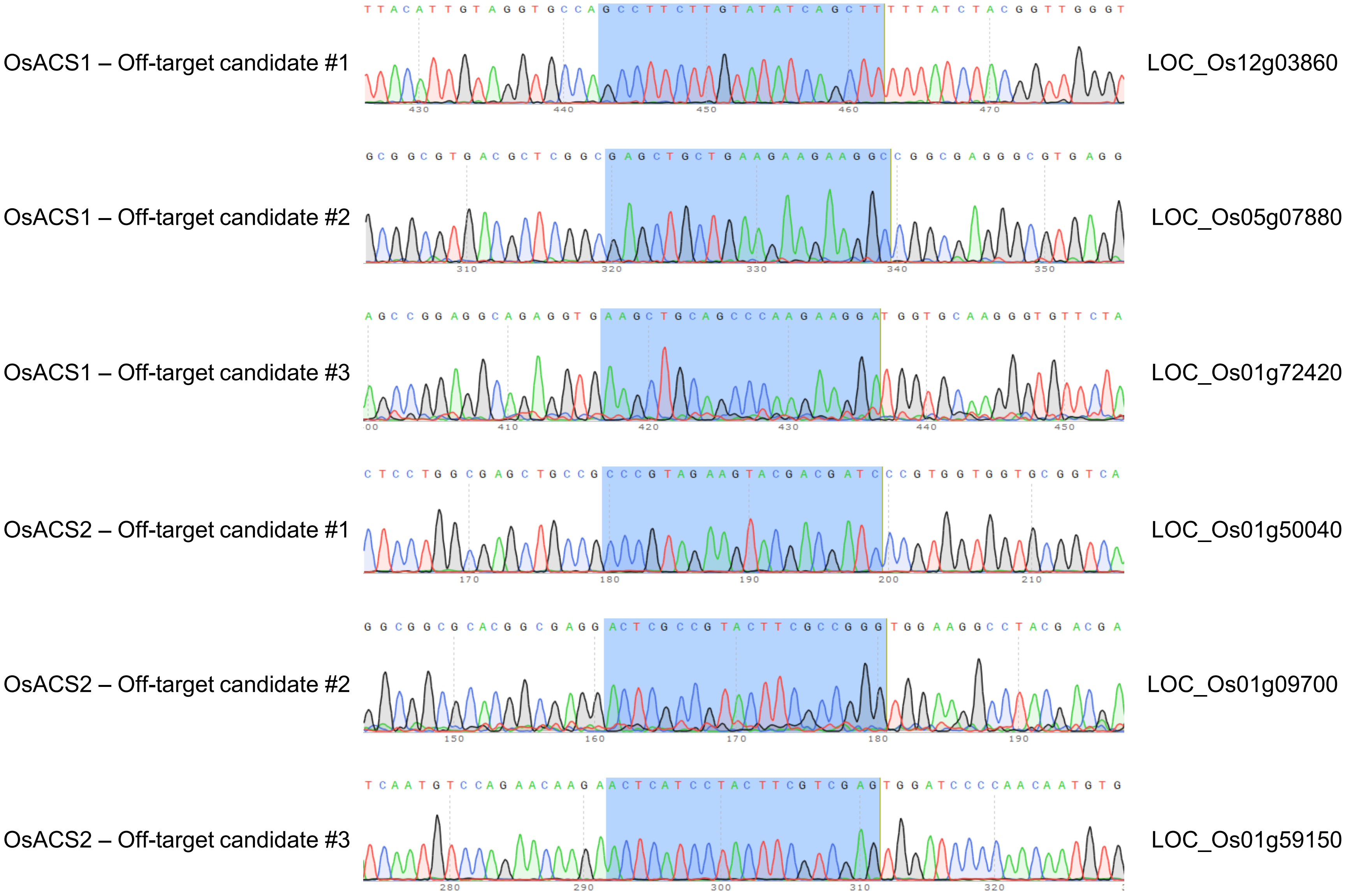

Fig. S5. Sequencing results of the potential off-target sites. The regions spanning the potential off-targets of *OsACS1* (LOC_Os12g03860, LOC_Os5g07880, and LOC_Os1g72420) and *OsACS2* (LOC_Os1g50040, LOC_Os1g09700, and LOC_OS1g59150) were PCR amplified from the genomic DNA of homozygous T2 Cr-*OsACS* mutants and sequenced.

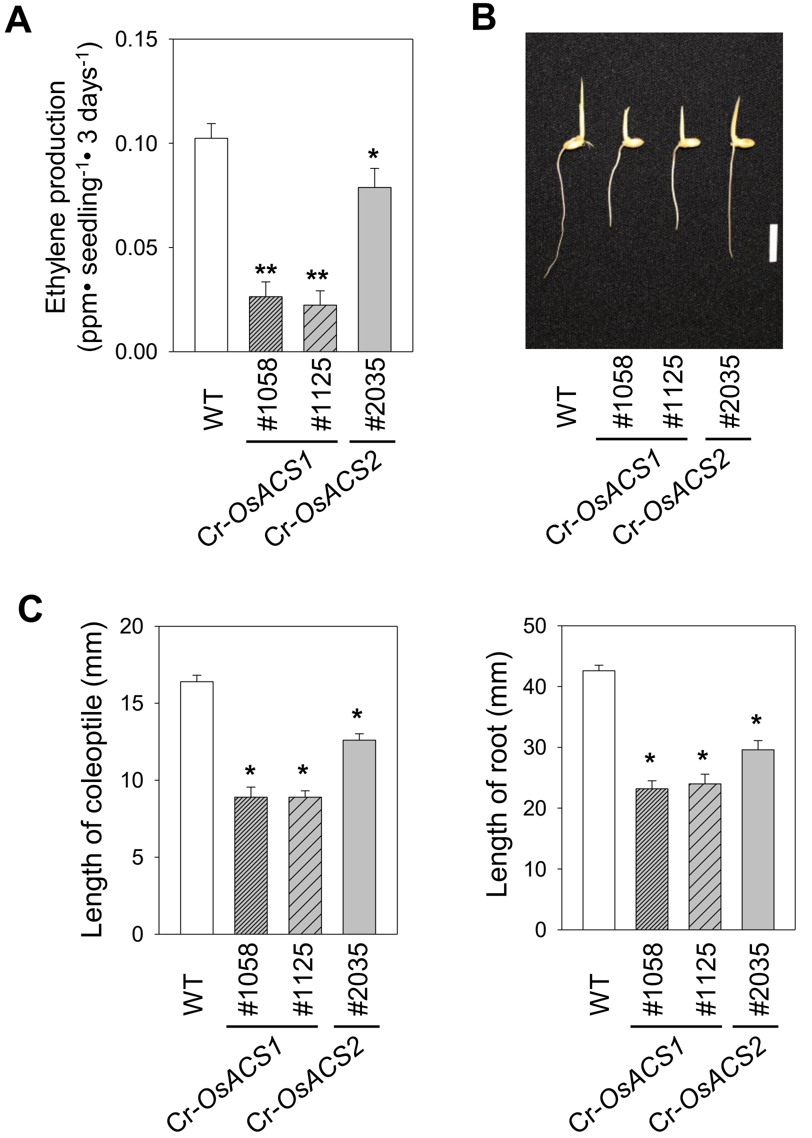

Fig. S6. Morphological analysis of Cr-*OsACS* T3 mutants and ethylene biosynthesis in the mutants. (A) Ethylene production of 3-day-old dark-grown WT and Cr-*OsACS* T3 mutant seedlings. (B) A representative image of dark-grown Cr-*OsACS1* (#1058 and #1125) and Cr-*OsACS2* (#2035) T3 mutant seedlings. Scale bar indicates 10 mm. (C) Measurement of the coleoptiles and roots length of Cr-*OsACS1* and Cr-*OsACS2* T3 mutant seedlings. Error bars indicate SD; n=5; * $P < 0.05$, ** $P < 0.005$, Student's *t*-test.

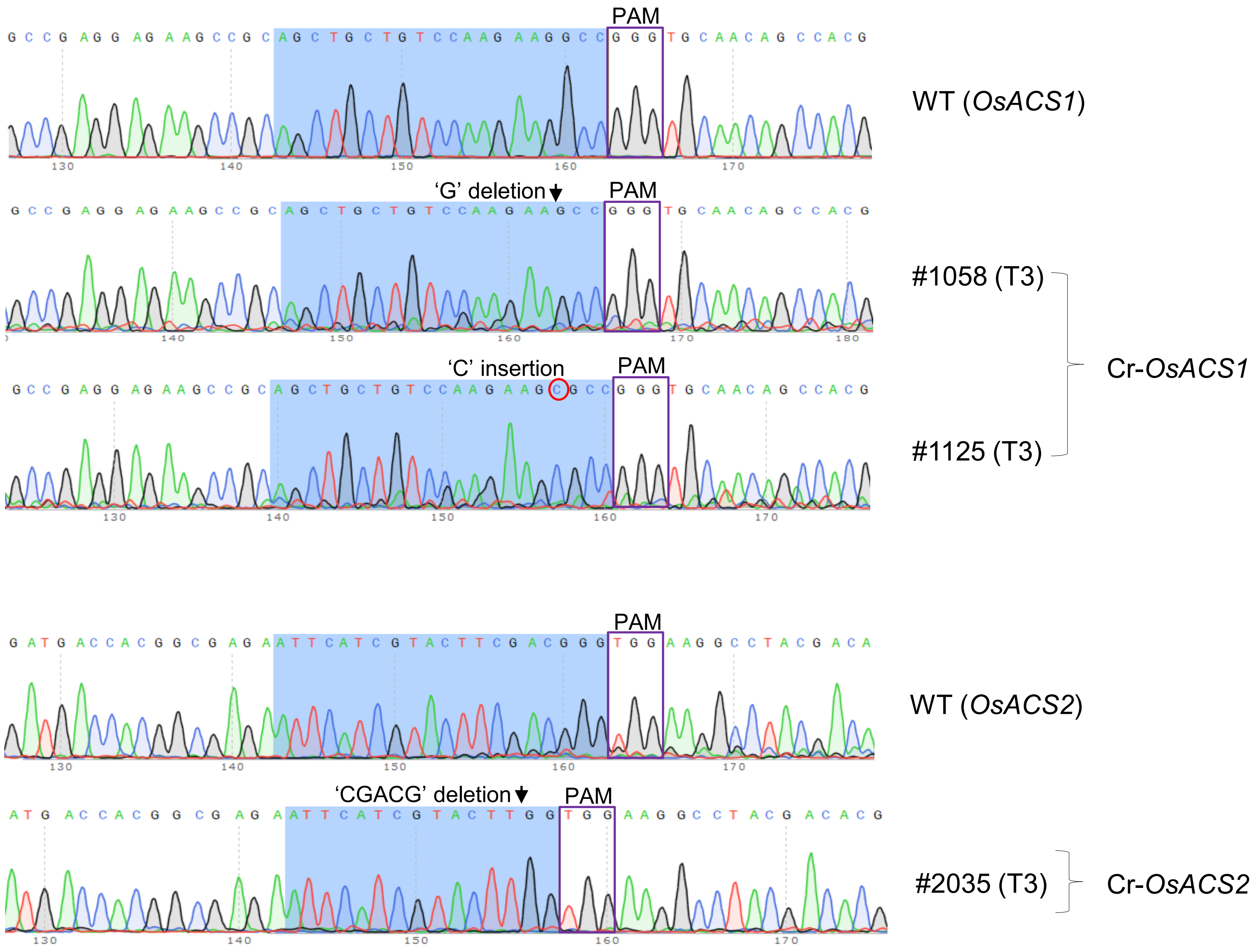

Fig. S7. Sequencing results of the T3 generation of Cr-*OsACS* mutants. The genomic DNA of wild-type and T3 lines of Cr-*OsACS* mutants without *Cas* were used for Sanger-sequencing analysis. The regions spanning the target sites of *OsACS1* (#1058 and #1125) and *OsACS2* (#2035) were PCR amplified and sequenced. A 1-bp deletion or 1-bp insertion was detected in the Cr-*OsACS1* (#1058 and #1125, respectively) and a 5-bp deletion was detected in the CR-*OsACS2* mutant (#2035). PAM sequences are squared with boxes.

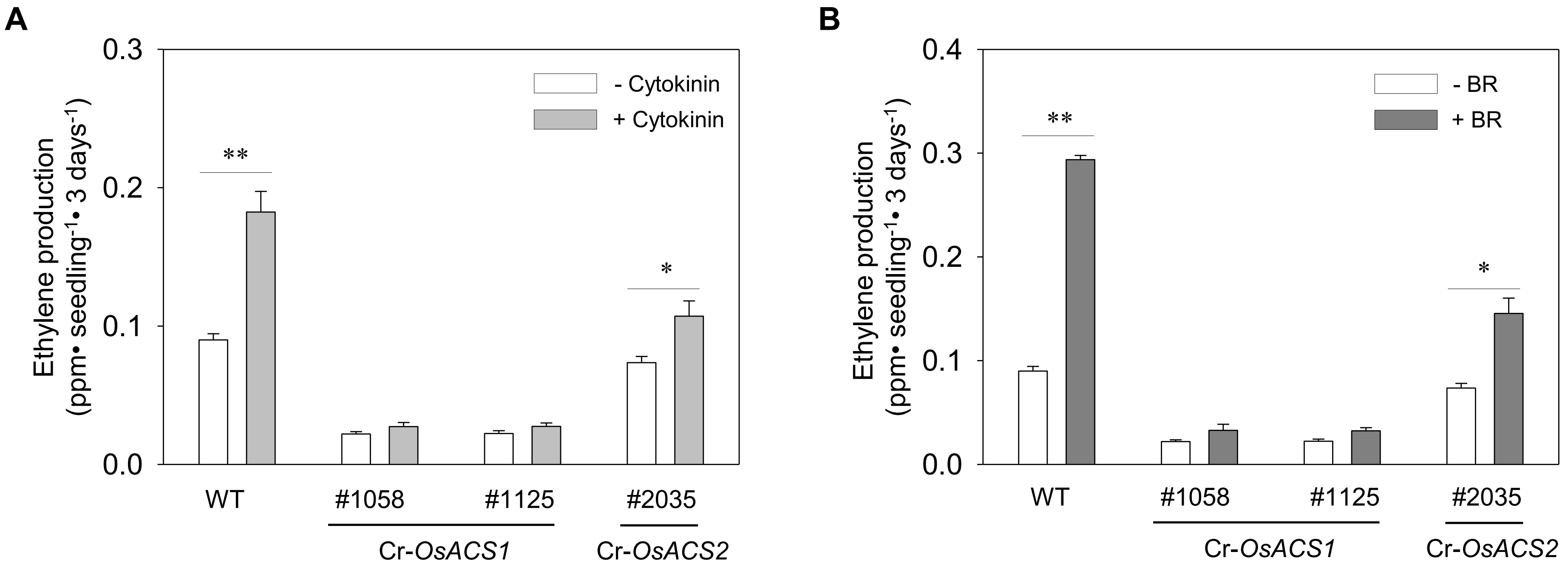

Fig. S8. Cr-*OsACS* mutants have impaired hormone-induced ethylene biosynthesis. (A, B) Cytokinin (A) or Brassinosteroid (B) -induced ethylene biosynthesis in 3-day-old dark-grown wild-type and Cr-*OsACS* mutant seedlings. Seedlings were grown in capped GC vials with (+) or without (-) 1 μM cytokinin or BR for 3 days and the accumulated ethylene was measured on day three. Error bars indicate SD; n=3, * $P < 0.05$, ** $P < 0.005$, Student's *t*-test.

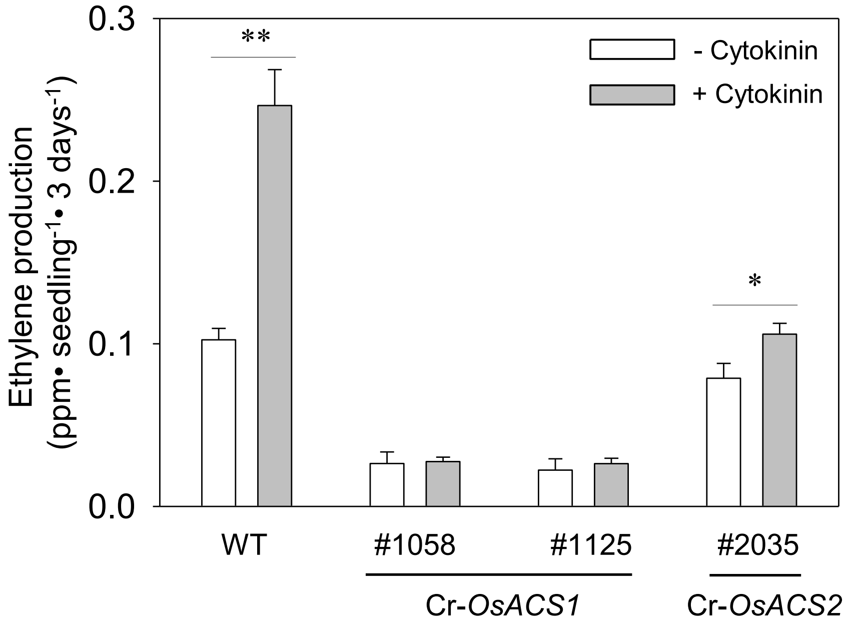

Fig. S9. T3 generation of Cr-*OsACS* mutants have impaired cytokinin-induced ethylene biosynthesis. Cytokinin-induced ethylene biosynthesis in three-day-old dark-grown wild-type and T3 lines of Cr-*OsACS* mutant seedlings. Seedlings were grown in capped GC vials with (+) or without (-) 1 µM cytokinin for three days and the accumulated ethylene was measured on day three. Error bars indicate SD; n=5, * $P < 0.05$, ** $P < 0.005$, Student's *t*-test.

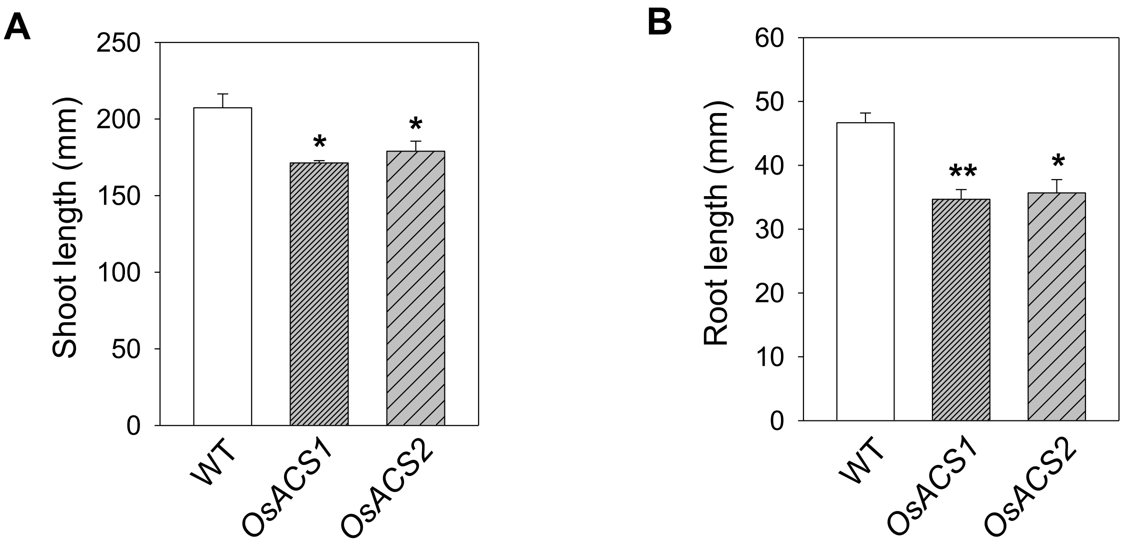

Fig. S10. Altered morphology of Cr-*OsACS* mutants in Pi-sufficient condition. (A, B). Seven-day-old light-grown seedlings were grown under Pi-sufficient condition. Statistical analysis of shoot (A) or root (B) length of WT and Cr-*OsACS* mutants. Error bars indicate SD; n=3. * $P < 0.05$, ** $P < 0.005$, Student's *t*-test.
