# Supplementary material for: Editing of the OsACS locus alters phosphate deficiency-induced adaptive responses in rice seedlings
Source: J Exp Bot. 2019 Feb 27;70(6):1927–40. doi: 10.1093/jxb/erz074 (PMC6436150; doi:10.1093/jxb/erz074)
Supplement: Supplementary Table S1 [file erz074_suppl_supplementary_table_s1.pdf]

Table S1. The list of primers used in this study.

Gene	Primer Sequence	
	Forward (5' to 3')	Reverse (5' to 3')
**For gene editing confirmation**		
OsACS1-genome	CTCCTACTCAGCAGCAGCAGCA	GTAGTCCTGGAACAGCGCGA
OsACS2-genome	GTCCGATCATCCTTGCGTGGCTA	ATTGGCCTTACCTTTCTGAACTCC
**For qRT-PCR**		
OsACS1	GCCTCTTCTGCTGGGTGGAC	ACATGTTGGCGAAGCAGACG
OsACS2	CGCGTCGGCATCATCTACTC	CGCCACCAGCCGACACTT
OsACS3	GGGCCTTGCAGAAAACCAG	CGCCTTGCCTCCCCTTATC
OsACS4	AAGGCGATGGCAAGATTCA	GCCCGTCCTCCACCTCAG
OsACS5	GCCGGTAGCTTCAGGGACAA	GGCGAGGATGAAGGTGAGGA
OsPT2	GACGAGACCGCCCAAGAAG	TTTTCAGTCACTCACGTCGAGAC
OsPT3	TGCGACTGCTGTATTCAGTACGT	ACAAATGCCATCAAATATGAACAGA
OsPT6	TATAACTGATCGATCGAGACCAGAG	TGGATAGCCAGGCCAGTTATATATC
OsSPX1	ACATTTGCTGGAGATAGTACCG	TCTGATGGTTATGATGGGTTTCT
OsSPX3	TGCCGGTACTAGATTGTATGATT	GTATGTTCTCTACCACGGCATA
OsIPS1	CTAAGGTAGGGCAACTTGTATC	TTATTAGAGCAAGGACCGAAAC
OsPIN2	TCTTGCATGTTGCCATTGTT	AACCGCTGTGCTAAGTATTT
OsPIN10a	TGACACGCCTCATCCTCATA	GATGGACTTCTCGACGATTG
OsIAA1	GACAAGTTCTTCTCCCACTTCACCA	CCTCATTGAGCGGCTCTTGGTG
OsIAA8	TTCCCTCACGACGGCGACAG	ATGATGAACCTTGTTCGTCTTC
OsARF1	GGAGGACGCGCTGTTCAC	ATCCGCTGCTCGCCTACCTG
OsYUCCA4	CCTCGACCTCTGCAACCACAAT	CAGGCGGGCCGTATCACC
OsAct1 (internal control)	TCATAGGAATGGAAGCTGCG	AGGAGCCAAGGCAGTGATCT
**For off-target confirmation**		
LOC_Os12g03860	GCATTTGCTGTAATAACCGTTTCA	TAATTGGTACAGATAGTGCCTGC
LOC_Os05g07880	CAAGGTCCACGTCAAGCTCCAGT	ACGATCTTCTGGTGGTGCGTG
LOC_Os01g72420	GACGACGACGACTGCGAGAT	AGCTCTGCTTCCGTAGAAC
LOC_Os01g50040	ATGCCGGCGGCGAAGAGGT	CGCTGGTTCGCTGGATCCTCGAG
LOC_Os01g09700	ATGGGGCGTGGCCAATTGC	TCCGGGTGGTCACGGAGGTAG
LOC_Os01g59150	GATTTCTGCCACCATGAGTGGA	TGAACATAGCAGTGAACTGCTC
